# Physicochemical modeling of electron FLASH to identify the roles of oxygen, aqueous electrons and causal radical recombination effects underlying them

**DOI:** 10.1088/1361-6560/aeabe5

**Published:** 2026-10-16

**Authors:** William S Thomas, Xu Cao, David J Gladstone, Harold M Swartz, Brian W Pogue

**Affiliations:** 1 Department of Medical Physics, University of Wisconsin School of Medicine and Public Health, Wisconsin, WI 53705, United States of America; 2 Thayer School of Engineering at Dartmouth, Hanover, NH 03755, United States of America; 3 Department of Radiation Oncology and Applied Sciences, Geisel School of Medicine at Dartmouth, Hanover, NH 03755, United States of America

**Keywords:** UHDR, FLASH, ROS, oxygen, simulation

## Abstract

*Objective.* Ultra-high dose-rate (UHDR) irradiation reduces normal-tissue toxicity as compared conventional dose-rate irradiation, known as the FLASH effect, yet the underlying radiochemical mechanisms remain poorly understood. Direct measurements rely upon longer lived water-radiolysis species such as aqueous electrons (e_aq_^−^) and oxygen, to infer behaviors of toxic species leading to tissue damage. This study uses kinetic modeling to identify which chemical pathways exhibit dose-rate-dependent behavior and can be used to correlate experiments. *Approach.* A comprehensive physicochemical model of water radiolysis was developed, incorporating 16 molecular species and 56 coupled reaction rate equations, with pulsed-beam structures matched to published UHDR electron irradiation experiments. Simulations were performed across a range of oxygen tensions (0.1%–10% O_2_) and dose-rate conditions (0.3–300 Gy s^−1^). Model predictions were validated against experimental measurements of e_aq_^−^ transients, and oxygen-consumption *in vitro* and *in vivo*. They also were compared to prior computational models. *Main results.* Modeled lifetimes of e_aq_^−^ matched experiments across O_2_ concentrations and showed that >90% of scavenging occurs via O_2_ and reactions with proteins, with radical–radical recombination contributing <5% even at 300 Gy s^−1^. The lifetimes of OH· radicals were similarly dominated by protein scavenging, with recombination pathways remaining minor (<5%) with UHDR. Modeled oxygen consumption matched the magnitude and dose-rate dependence observed in published experiments. The results indicated a primary dose-rate sensitive pathway, the reaction between hydroperoxyl radical (HO_2_·) and superoxide (O_2_·^−^). Peroxyl-radical formation decreased at UHDR within oxygen tensions (≈5–20 mmHg) corresponding to the experimentally observed FLASH oxygen-window. *Significance.* This work identifies specific radiochemical pathways that exhibit true dose-rate sensitivity and clarifies which proposed FLASH mechanisms are unsupported by homogeneous-phase radical kinetics. Radical–radical recombination contributes too little (<5%) to account for known FLASH sparing at UHDR, whereas peroxyl-radical and HO_2_·/O_2_·^−^ chemistry shows dose-rate-dependent behavior within physiologic oxygen tensions associated with FLASH. Although constrained by simplified aqueous conditions lacking scavengers and tissue heterogeneity, the model provides mechanistic insight into the radiochemical regimes dominating the homogeneous phase chemistry.

## 1. Introduction

Over the past decade, substantial experimental and theoretical efforts have focused on producing and characterizing the FLASH effect, which reduces radiation-induced normal tissue damage when ultra-high dose rate (UHDR) is used, while retaining comparable tumor control in preclinical models. This FLASH effect appears modality agnostic, having been demonstrated with electrons (Favaudon *et al* 2014), photons (Montay-Gruel *et al* 2018), protons (Singers Sorensen *et al* 2022) and heavy ions (Tinganelli *et al* 2022) suggesting that the underlying mechanisms are rooted in fundamental radiation chemistry and/or radiobiological processes. This rapidly growing field of study is at the nexus of radiation therapy, radiobiology, radiation chemistry, and radiation physics all required to determine the origins of this effect. Although the underlying mechanism remains unknown, the potential for human use has resulted in several clinical studies (Mascia *et al* 2023, Kinj *et al* 2026). The most basic mechanisms in radiation chemistry from UHDR are challenging to investigate definitively, yet it seems very likely that the origins of the FLASH effect come from the ultra-fast delivery of radiation, stimulating a change in the resulting radiation chemistry cascade. In this study, we use a detailed kinetic model of radiolysis to interrogate the chemical interactions most likely to govern dose rate-dependent responses, with an emphasis on experimentally measurable outcomes such as oxygen consumption dynamics and kinetics of the hydrated electron.

Despite enormous growth in experimental studies, the mechanism behind the FLASH effect remains with a variety of divergent hypotheses, which still seem to have no clear convergence. Oxygen consumption and radical–radical recombination have been leading candidates as the species change to cause the FLASH effect. Oxygen consumption was initially thought to have been severe enough to induce transient hypoxia, at the level of reducing the relative biological effect, thereby being damaged less. This would contrast with the lack of change from oxygen consumption in the already hypoxic tumor tissue (Adrian *et al* 2020, Cao *et al* 2021). An alternate hypothesis was that radical–radical recombination is occurring with increased hydroxyl radical density being created locally, which would lower the resulting amount of DNA damage, and this agrees with experimentally observed data. Also there is an observed significant reduction (∼30%) of hydrogen peroxide yield at UHDR, indicating that the cascade of radiation chemistry occurring clearly must be changing with dose rate (Montay-Gruel *et al* 2019, Kusumoto *et al* 2024). However, no study has exclusively linked these changes directly to the biological FLASH effect, and theories have continued to emerge and evolve including redox metabolism, immune modulation, vascular preservation (Limoli and Vozenin 2023).

Direct tests of the fast radical–radical recombination effects that are hypothesized by some to underlie the FLASH effect are constrained by detection limits, due to the short lifetimes of key reactive oxygen species (ROS) such as hydroxyl radicals (OH·). This logistical problem forces many studies to infer the radiation chemistry from stable or longer-lived products, rather than the short-lived reactants themselves. Measurements of ROS in biological media often use passive monitoring of long-lived ROS species such as hydrogen peroxide via fluorescent optical probes (Montay-Gruel *et al* 2019, Sunnerberg *et al* 2023a, 2023b) due to these limitations. In this context, computational kinetic modeling provides critical access to otherwise unresolvable molecular time courses and reaction fluxes.

After the initial very short-lived products from the interaction of ionizing radiation, there are two primary products that can diffuse and damage relevant biological molecules, the aqueous electron and the hydroxyl radical with lifetimes on the order of microseconds in pure water to sub-nanoseconds in complex biological systems. Hydroxyl radicals are thought to be the leading cause of DNA damage (Halliwell *et al* 2021) while aqueous electrons (e_aq_^−^) are rapidly scavenged by oxygen to create superoxide (Laverne and Pimblott 1993). Although e_aq_^−^ is less directly cytotoxic than OH·, it participates broadly in the subsequent reaction cascade which could impact cytotoxicity. Also, it is one of the few reactive species that can be experimentally monitored (Thomas and Hart 1964, Elliot 1989, Megroureche *et al* 2023, Cao *et al* 2025, 2026) in real time due to its strong optical absorption signature. Our model aims to explicitly quantify these transient interactions and test which reactions contribute to dose rate-dependent chemistry, under relevant oxygen tensions and with varying linac pulse lengths.

Computational kinetic modeling can provide time-resolved prediction of the short lifetime ROS species that lack sufficient observable features. Extensive work has been done in modeling of the earliest phases of radiation tracks and the resulting inhomogeneous phase (Boscolo *et al* 2021, Chappuis *et al* 2023, JN *et al* 2025) and multiple approaches to physiochemical modeling have been developed to evaluate how UHDR alters radiolysis products and their subsequent interactions with oxygen, DNA, proteins, and lipids (Bielski *et al* 1985, Buxton *et al* 1988, Neta *et al* 1990). There is general agreement that the final phase of ROS cascade can be effectively modeled as a homogeneous phase of interactions between the primary radicals, oxygen, and downstream molecules such as proteins. In this study, we employ a detailed physicochemical rate model to quantify chemical reaction rate interactions and investigate how dose rate and oxygen influence radical production, specifically to compare the predictions with experimentally measured *in situ* data.

This study simulates the kinetic chemical model for UHDR founded on radiochemical kinetic theory as described by Larbarbe *et al* (2020) and Tan *et al* (2023), The goal here was to specifically to examine how oxygen variation affects the ROS cascade and to use this to match to experimentally measured data. Extensive simulations were carried out with validation done by comparison to other models, and to experimental data of oxygen consumption and hydrated electrons. The novelty of this study comes in the form of results of specific reaction rates at various dose rates and oxygen levels to characterize leading hypothesis of the FLASH effect.

## 2. Methods

### 2.1. Simulation

There are several temporal stages to water radiolysis from ionizing radiation, starting with the initial energy deposition and formation of spurs that give rise to more stable molecules and radicals. These processes occur on a very fast timescale but can lead to downstream radiolytic transient species that react on the order of nanoseconds to microseconds in a nearly homogeneous phase (Pelletier-baillargeon and Baillargeon 1965, Belloni *et al* 2008). During this stage, conventional chemical reaction rates are thought to describe the overall system, and nearly all rate constants have some measured estimate. In this study, the intended focus was on those species and the resulting reaction rate processes that are key to damage of DNA and thus lead to the killing of cells.

This model follows reaction rate kinetics through ordinary differential equations (ODEs) describing early radiation-initiated creation of radiolytic species created from a 9 MeV electron beam as seen in table 1. This focuses on a chemical timescale of 10^−10^ s to 100 s for all simulations. While earlier attempts (Labarbe *et al* 2020) have focused on just nine molecular species in the rate model, here 16 distinct moieties were included and modeled over time (H_2_O, O_2_, OH·, H·, OH·, H_2_O_2_,H_2_, O_2_–, OH−, HO_2_·,HO_2_–, O·, O_3_, H+, R·, ROO·) where R denotes a generic carbon-centered molecule with reaction rates close to albumin, often measured in bovine serum albumin (BSA) when available in literature. These molecular species are then tracked through a set of coupled nonlinear first-order equations encapsulating 56 different reaction rate equations (Supplemental material 1) and iterated over a fixed period of time. The primary yield values for the specific irradiation modality of 9 MeV electrons in solution of pH at 7 are shown in table 1 and are defined from literature as developing 1 us after a pulse. Initial concentration levels of O_2_ simulated from 6–124 *µ*M (0.1%–10%, 0.76–76 mmHg), with 602 *µ*M RH. These yields were used for simulation. With simulated examples of protein solutions in figure 1 where total dose of 10 Gy was delivered by UHDR or conventional dose-rate (CDR).

**Figure 1.**
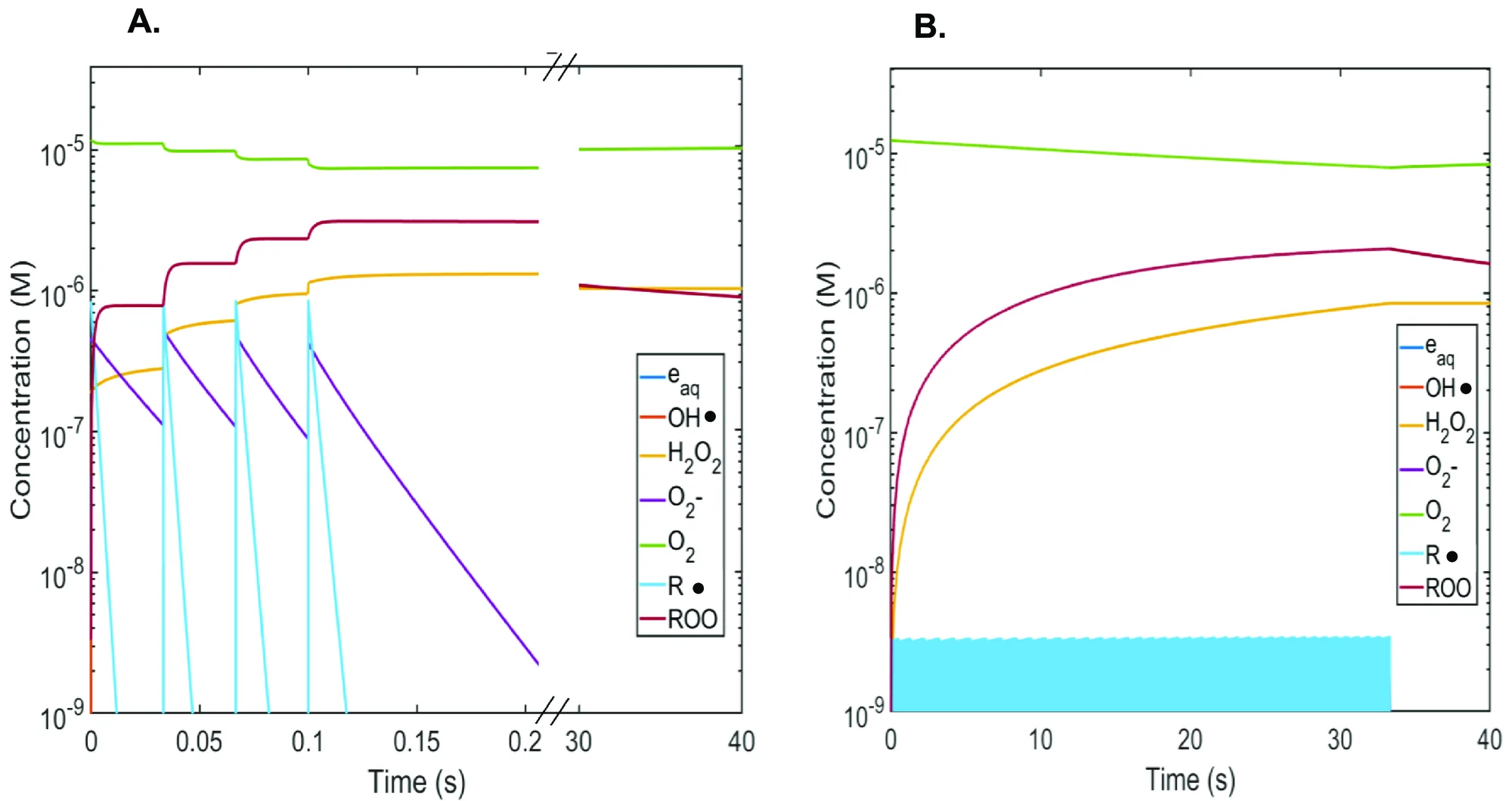
Example of UHDR (75 Gy s^−1^ Avg DR/0.65 MGy s^−1^) (a) and CDR (0.1 Gy s^−1^) (b) simulations with several key radiolysis species plotted through to equivalent steady state. Initial O_2_ levels for these examples are 1%. All simulations were carried out to 100s, to allow decay of peroxyl radicals.

**Table 1.** The concentration yields of radiation induced products of radiolysis from ∼9 MeV electrons, for solutions with a pH of 7 (Boyd *et al* 1980, Frongillo *et al* 1998).

Product	e_aq_^−^	·OH	H·	H_2_O_2_	H_2_	OH−	H+	R·
Units (*µ*M Gy^−1^)	0.27	0.278	0.06	0.07	0.05	0.01	0.28	0.06

### 2.2. Radiation source

With the goal of validating the model to experimental data (Cao *et al* (2025), Sunnerberg *et al* (2025)), we used a pulsed beam structure into the radiation source for the reaction kinetics ODEs with an MeV electron linac source for our modeling. The linac used was a FLASH Mobetron (IntraOp Inc. Sunnyvale CA) which had pulse lengths of 4 us, and a pulse repetition frequency of 30 Hz. The dose per pulse was varied by modifying overall total number of pulses beginning at a singular pulse of 10 Gy. To vary the dose rate in these simulations, the amplitude of the pulse was lowered inversely to the number of pulses beginning from one singular pulse of 10 Gy to 1000 pulses of 0.01 Gy. This range of dose per pulse corresponds to average dose rates of 0.3–300 Gy s^−1^, with corresponding instantaneous dose rates of 2.5 kGy s^−1^–2.5 MGy s^−1^.

### 2.3. Oxygen measurement & consumption

The experimental setup for varying oxygen is described in previous studies (Cao *et al* 2021, El Khatib *et al* 2022, Sunnerberg *et al* 2023b, 2025) and measuring it was based upon the molecular quenching of phosphorescence lifetime of Oxyphor, dissolved in aqueous media or *in vivo*. The measurement uses a pulsed LED fiber coupled device to measure the emission lifetime, which provides calibrated absolute oxygen values and samples data sufficiently fast to measure both steady state and transient oxygen values in FLASH studies. The *in vitro* experimental measurements were done in 1 cm square cuvettes, mixed in pure water with 4% (600 *µ*M) BSA, with Oxyphor probe at 2 *µ*M. These experimental parameters were used for comparison in this study to UHDR of 300 Gy s^−1^ and CDR of 0.1 Gy s^−1^ from Cao *et al* (2021) and Sunnerberg *et al* (2023b).

### 2.4. Aqueous electron measurements

The experimental setup for measuring hydrated electrons was described in previous studies (Cao *et al* 2025, 2026) using transient absorption spectroscopy to quantify the relative magnitude produced under different dose, dose rate, and oxygen conditions. Measurements were performed in a quartz cuvette with dimensions 45 × 12.5 × 102.5 mm, filled with 35 ml of pure water. A 4.5 MW laser diode emitting at 670 nm wavelength (CPS670F, Thorlabs, New Jersey, USA) was directed through the cuvette, using a multi-pass technique to increase the path length. The multi-pass system was composed of two dielectric broadband mirrors on either side of the cuvette, resulting in a total path length of 110 cm. The laser beam was then detected after passing through a long pass filter (cutoff 650 nm) to minimize contaminating signals from Cherenkov emission and room lights. The light signal was collected by a silicon photodiode (model DET10A2, Thorlabs, New Jersey, USA) with an active area of 0.8 mm^2^ and a temporal response of 1 ns. The detected signal was amplified by an oscilloscope (model 5442D, Pico Technology Ltd, Cambridge shire, UK) with a sampling rate of 62.5 MHz, with data recorded by MATLAB. The cuvette was positioned fully within the radiation field, while the photodiode and remaining electronics were positioned over 2 m away to minimize electronic noise. The transients of the signal measurement were recorded with the linac beam current transformer to map when the pulses were activated and measure absorption during the beam on to quantify aqueous electron yield. UHDR radiation was provided by a FLASH Mobetron unit which delivered a 9 MeV electron beam, using 4 us pulse lengths and varying average dose rate either through pulse repetition frequency and source to surface distance (SSD), or only instantaneous dose rate via SSD. Beam dose delivery was recorded via EBT-XD Gafchromic film placed above and below the glass cuvette with the dose averaged between the two points (Ashland, Bridgewater, NJ). In order to measure the delivered dose in film, a calibration curve was created by exposing films to known doses in solid water on a Varian TrueBeam (Varian Medical Systems, Palo Alto, CA) LINAC at an equivalent energy of 9 MeV. Irradiated film was placed onto a plastic holder to ensure identical film orientation for scanning both pre and post radiation on an Epson Expression 10 000 XL (Epson, Los Alamitos, CA) with changes in optical density compared to both calibration films and known optical density filters to establish dose delivered.

## 3. Results

### 3.1. Radical recombination effects

We compared the lifetime of aqueous electrons at 1% and 10% oxygen in pure water, which is relevant because oxygen is the primary scavenger for aqueous electrons when oxygen is present. At lower oxygen concentrations reactions with other components of cells can occur. High oxygen concentration leads to a lower aqueous electron lifetime, as seen in figure 2(b) versus a. Overall mean lifetimes of the experimental and simulated data are quite similar at lower oxygen with a lifetime of 2.4 and 2.7 us respectively. At 10% oxygen the lifetimes are, 0.29 us experimentally and 0.4 us in simulations. The results show substantially similar behavior both during the pulse and in the decay of aqueous electrons after the pulse ends for both low and high oxygen levels. Because aqueous electrons are involved in recombination reactions, this provides first level correlation evidence that the model predicts what is occurring experimentally.

**Figure 2.**
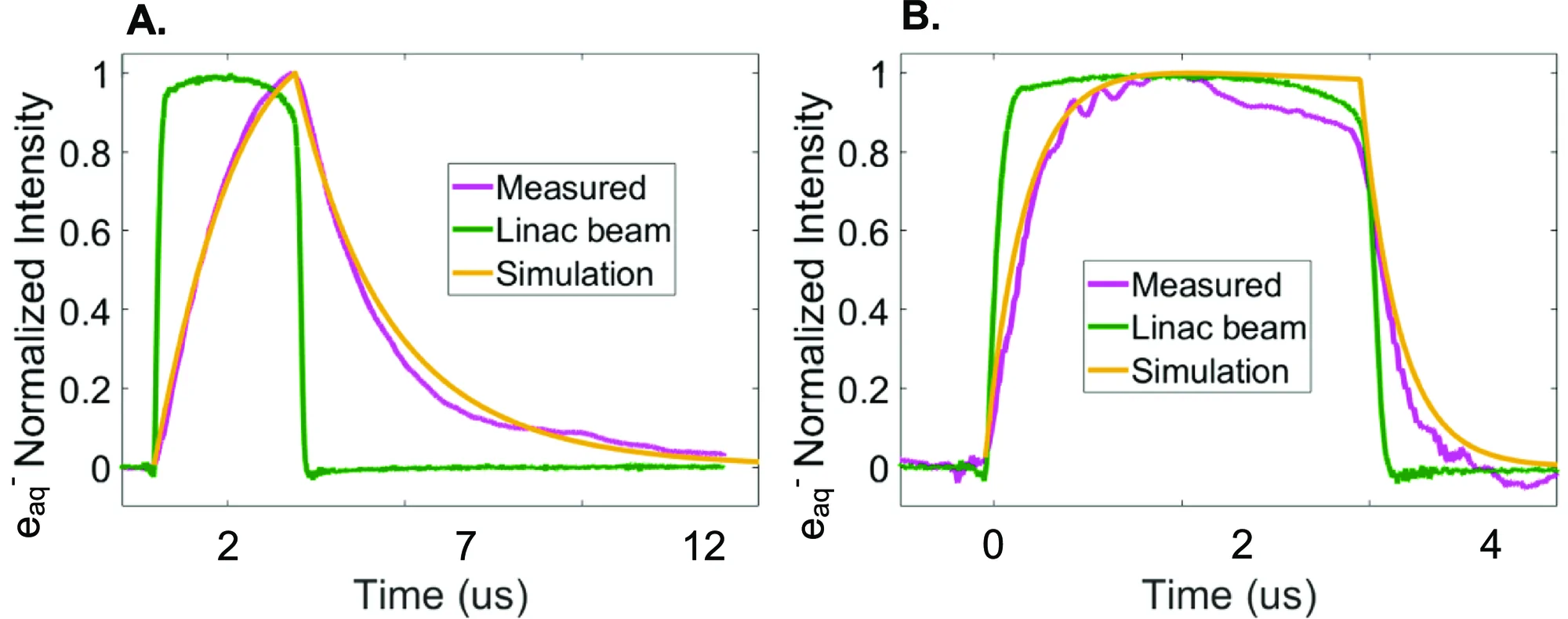
Measured experimental data and simulated rate equation data for transient aqueous electrons e_aq_^−^ from a single 4 *μ*s UHDR pulse at both low (∼1%) oxygen (A) and high oxygen (∼10%) (B). The measurements (pink) are overlaid with the simulations (yellow) and the pulse shape of the beam (green).

As the simulated time evolution of aqueous electrons is substantially similar to the experimental data, we further examined which reaction rate equations were contributing to the overall scavenging of aqueous electrons. This was done in a protein rich solution with the O_2_ level set to 5%. From these simulations we can see which reaction rate equations are key in scavenging aqueous electrons. Approximately 90%–95% of aqueous electrons generated by the primary beam are scavenged by oxygen (∼80%–85%) and RH (10%) as seen in figure 3. At the highest simulated dose rate, where all 10 Gy are delivered in one 4 us pulse, one can see the reaction of aqueous electrons and hydroxyl radicals begin to occur at a noticeable yield accounting for ∼2% of overall scavenging. This recombination effect of 2% is only noticeable at dose rates well above the commonly established FLASH effect level of ∼40 Gy s^−1^.

**Figure 3.**
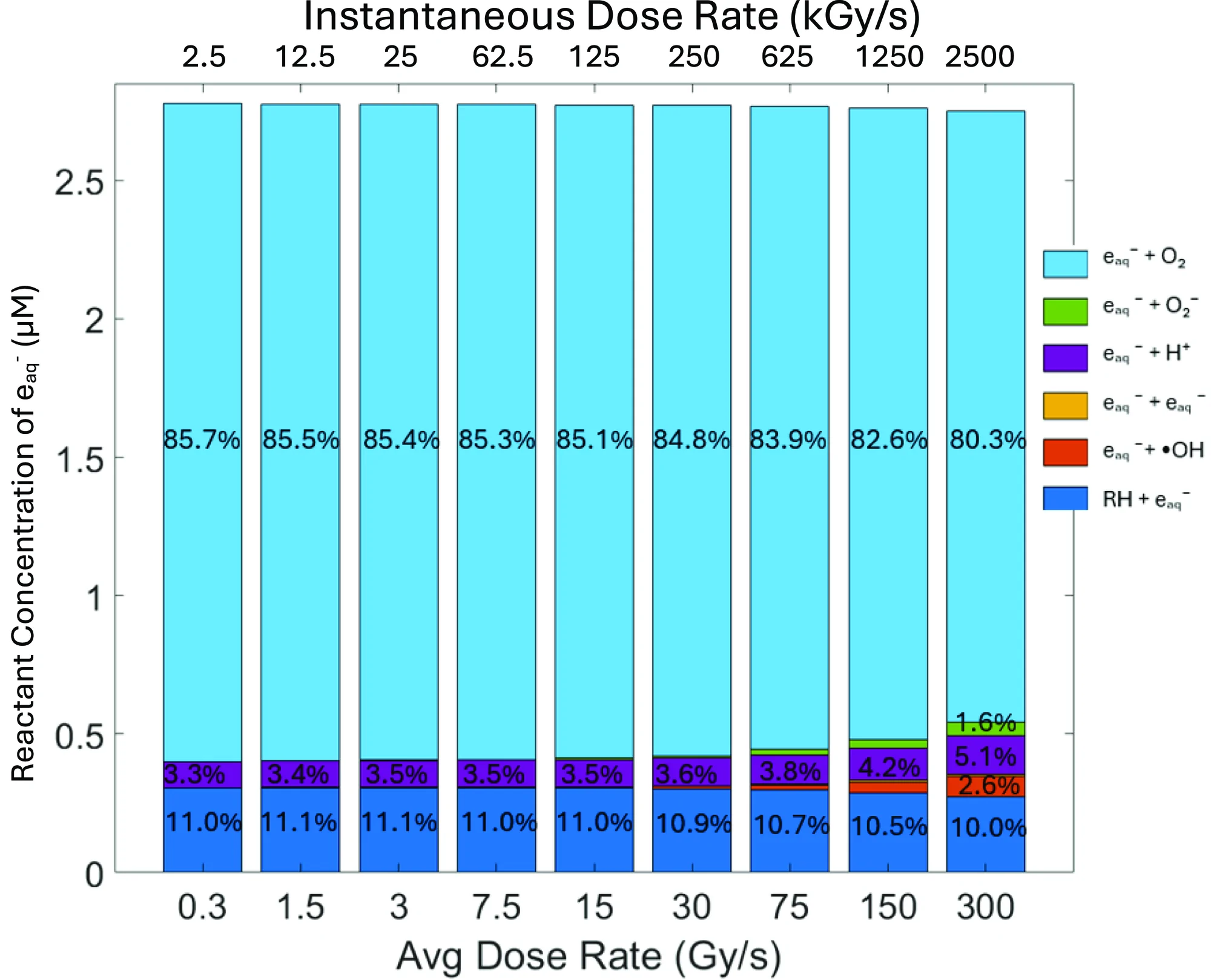
Hydrated electron scavenging reaction yields from simulated data using the following parameters: Initial O_2_: 5%, pulse repetition frequency: 30 Hz, total dose 10 Gy, pulse width: 4 us.

When applying this same method to the more biologically damaging hydroxyl radicals, a similar result can be seen in figure 4. Hydroxyls are primarily reactive with the protein in solution (∼95%–99%), with minor amounts (<5% total) of scavenging occurring from recombination with hydroxyl or a reaction with aqueous electrons.

**Figure 4.**
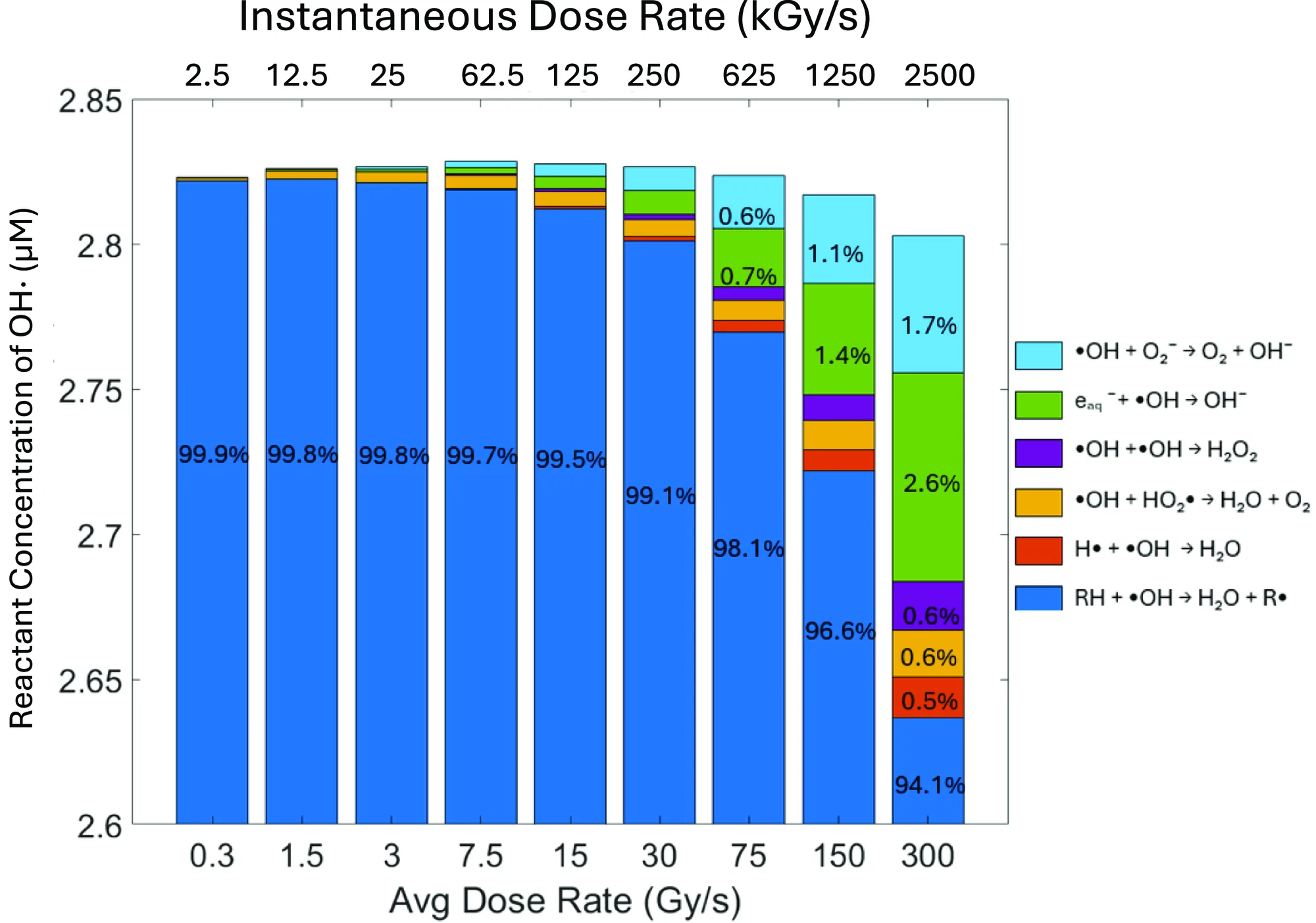
Hydroxyl radical scavenging reaction yields from simulated data using the following parameters: Initial O_2_: 5%, pulse repetition frequency: 30 Hz, total dose 10 Gy, pulse width: 4 us.

### 3.2. Oxygen consumption

Despite the fact that oxygen dominates radiation chemistry, determining if there is a causal role of oxygen in the FLASH effect has been inconclusive. Initially, it was proposed that depletion of oxygen during radiation pulses could cause transient hypoxia thereby reducing radiobiological damage by exploiting the reduced oxygen enhancement ratio. While several *in vitro* (Van Slyke *et al* 2022) and *in vivo* studies have shown a significant dependence of the FLASH effect on oxygen, the doses used show insufficient consumption of oxygen to induce hypoxia that would cause sparing in this manner (Cao *et al* 2021, El Khatib *et al* 2022). The model by Tan *et al* (2023) shows a high level of agreement between two experimental data sets of both proton and electron oxygen consumption levels. To validate our models of oxygen consumption we compared our data to simulations by Tan *et al* (2023) and *in vivo* data from Sunnerberg *et al* (2025). Simulations of electron irradiations from Tan *et al* at similar concentrations of BSA varying from 2%–5% show ΔpO_2_ plateauing at 20 mmHg (∼3% O_2_) with values of ∼0.27 mmHg Gy^−1^ (*G*-Value 0.35 *µ*M O_2_/Gy) at CDR and 0.23 mmHg Gy^−1^ (*G*-Value 0.3 *µ*M O_2_/Gy) at UHDR. Although the magnitude of these values differs from our model, seen in figure 5, by ∼10% the overall trends are identical and the difference in magnitude is most likely due to the presence of scavengers such as Glutathione (GSH) in the model by Tan *et al* When compared to a fit from Sunnerberg *et al* (2025) *in vivo* data of oxygen consumption in murine skin at UHDR, plotted in figure 5, there is a similar plateau at oxygen levels above 30 mmHg (4%) O_2_ showing a similar overall behavior of oxygen consumption in tissue, while exact magnitude and slope behavior is most likely dictated by various proteins and scavenger combinations as described by Koch *et al* (2023).

**Figure 5.**
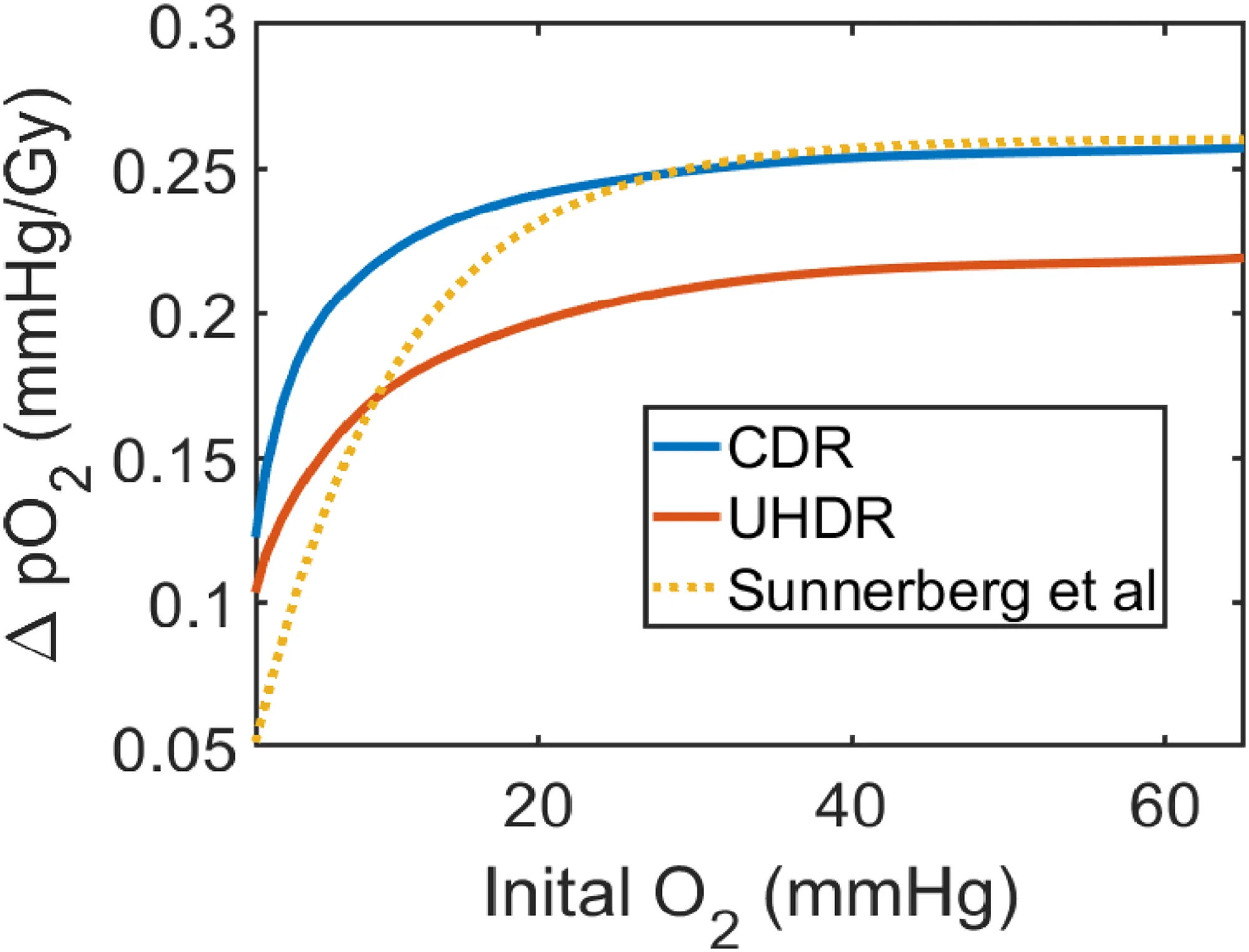
Comparison of oxygen consumption simulation at 150 Gy s^−1^ (UHDR) and 0.3 Gy s^−1^ (CDR) respectively, with *in vivo* data from Sunnerberg *et al* (2025).

With these comparisons in mind, we examined which reaction pathways in which oxygen is consumed via integrating this reaction in the protein solutions over time and compared them at UHDR (150 Gy s^−1^) vs CDR (0.3 Gy s^−1^). Figure 6 shows the 6 equations which contributed most to the overall change in O_2_, either by consuming (A–C) or supplying (D–F) oxygen to the system. Overall CDR and UHDR vary primarily at extremely low oxygen levels reserved for only <3 mmHg or 0.4% O_2_, while most equations reach unity when examining the ratio of UHDR to CDR of total yield plotted on the alternate *y*-axis. The primary contributor to the consumption or generation of O_2_ that varies between dose rates across all oxygen levels is the combination of a Hydroperoxyl radical (HO_2_·) with a superoxide (O_2_·^−^) which combine to form oxygen (O_2_) and a hydrogen peroxide anion (HO_2_−). This difference represented by the blue line in figure 6(D) shows a UHDR/CDR ratio of approximately 3 corresponding to a difference of an additional 0.4 *µ*M of superoxide and Hydroperoxyl reacting at UHDR. This results in an additional 0.4 *µ*M of oxygen or 0.31 mmHg of oxygen in the system and when normalized per unit Gy is 0.031 mmHg Gy^−1^ which accounts for nearly the entire difference in of the oxygen consumption observed in figure 5 of 0.25 mmHg Gy^−1^ at CDR vs 0.21 mmHg Gy^−1^ at UHDR.

**Figure 6.**
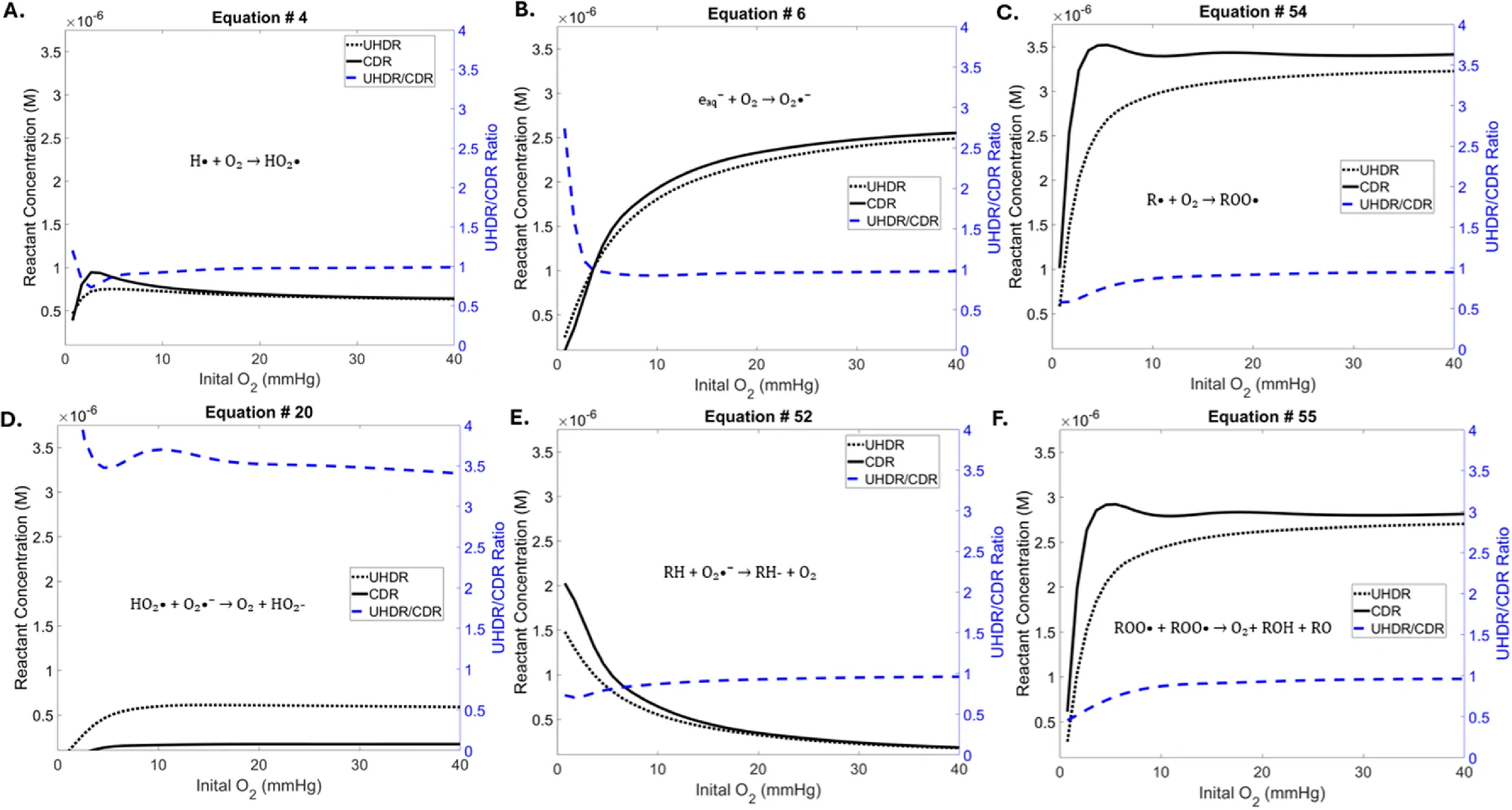
Dominant oxygen consuming reactions (A)–(C) and oxygen producing reactions (D)–(F) are shown as simulated for varying initial oxygen, with UHDR and CDR concentrations of each reactant (left axis) and UDHR/CDR ratio (right axis). Data was simulated using the following parameters: pulse repetition frequency: 30 Hz, total dose 10 Gy, pulse width: 4 us. UHDR (Dotted line) corresponds to 150 Gy s^−1^ Avg DR, 1.25 MGy s^−1^ Inst DR, 5 Gy per pulse delivery, and CDR (Solid Line) corresponds to 0.3 Gy s^−1^ delivery.

## 4. Discussion

This study used published literature based on kinetic reaction rates of molecules with simulations of pulsed irradiation at varying dose rates and oxygen levels to simulate the transients of all major reactive species via their systems of ODEs focusing on ∼9 MeV electrons at neutral pH (Boyd *et al* 1980, Frongillo *et al* 1998, Labarbe *et al* 2020). The study goal was to examine if these validate the trends in data from oxygen depletion in experimental studies in Sunnerberg *et al* (2025) and aqueous electrons produced by Cao *et al* (2025). The pulse frequency and pulse length simulated were chosen to match these experimental set ups, and the general trends were used as a metric of success of the simulation. This model examined the leading FLASH hypotheses, which are that radical–radical recombination and oxygen consumption changes occur with UHDR, and that this likely leads to lower radiobiological damage in normal tissues. By analyzing which specific reaction rates corresponded to trends validated in experiments, the goal was to identify the causal chemical reactions that underpin these observations.

**Hydroxyl ions:** Hydroxyl ions are critically relevant to the biological impact of radiation as shown by immense impact of reacting with proteins (95% of all Hydroxyl radicals) at all dose rates in this simulation. Perhaps one of the most interesting overall observations in this study is from figures 2 and 3, that the model predicts that overall radical recombination accounts for less than 5% of the scavenging of key products of radiolysis, for both aqueous electrons and hydroxyl radicals, even at UHDR up to 300 Gy s^−1^ Avg DR/2.5 MGy Inst DR The reaction with the largest dose rate dependence is the reaction between aqueous electrons and OH·, as seen in figure 3, where it goes from 0% to 2.6% of OH· reactions across the dose rate range simulated. This low level of change with dose rate suggests that radical quenching alone would have insufficient scavenging to reduce damage at the level seen in FLASH normal tissue sparing, which can be as high as 20%–30% damage reduction (Bohlen *et al* 2022). It is possible that scavenging is higher than this 5% level in the inhomogeneous phase of the reactions, where localized concentrations could be higher, however this is unknown and unvalidated at this time as it is faster than is possible to measure experimentally. Further Monte Carlo transient simulations would need to be completed to analyze this contribution (Abolfath *et al* 2020, Chappuis *et al* 2024).

**Aqueous electrons:** Aqueous electrons are like Hydroxyl radicals in terms of short lifetime, but react with a much wider array of species in radiolysis: O_2_, RH, H_2_O_2_, O_2_^−^, and OH·. The ability to monitor these aqueous electrons in real time gives a unique view into the behavior of short-lived species during UHDR radiotherapy and helps validate our model. Recombination is low in aqueous electrons accounting for less than 5% of electrons leaving the system at the highest simulated dose rate of 300 Gy s^−1^ Avg DR/2.5 MGy Inst DR The reaction with the largest dose rate dependence is the reaction between aqueous electrons and OH· as seen in figure 3 where it goes from 0% to 2.6% of overall aqueous electron reactions across the dose rate range simulated. It is well known that O_2_ is the primary scavenger of aqueous electrons this model agrees, showing at physiological O_2_ of 38 mmHg (5% O_2_) over 80% of aqueous electrons are scavenged by O_2_ and converted into O_2_^−^ regardless of dose rate. The abundance of aqueous electrons reacting with superoxide in a short period of time at UHDR is the likely cause of downstream oxygen changes due to combination of hydroperoxyl and superoxide as noted in figure 6.

**Oxygen:** The analysis of oxygen consumption is important, because of the growing amount of data showing how *in vivo* oxygen levels affect the observation of the FLASH effect, such as in normal skin sparing (Adrian *et al* 2020, Hunter *et al* 2025, Sunnerberg *et al* 2025). Several recent studies point to a window of tissue oxygenation required to illicit normal tissue FLASH sparing, specifically at concentrations below 20 mmHg O_2_. This is important but does not fully explain why FLASH is seen in brain, colon, or lung, where the normal tissue oxygenation will vary from skin (Grilj *et al* 2024). There is also an observation that oxygen consumption *in vitro* is lower at UHDR than at CDR, implicating a possible role of reduced ROS yields at UHDR, and therefore possibly reduced DNA damage at UHDR vs CDR. The underlying chemistry leading to these effects is not well described, and it is unclear if there is a causal relationship between these effects and FLASH normal tissue sparing. Thus, simulations as a function of oxygen were a particular focus in this work.

The trend of the model data generated in figure 5 shows oxygen consumption values to be consistent in magnitude and trend with baseline oxygen, comparing experimental data and prior simulation results. This provides confidence in the model for quantitative estimation of oxygen consumption. With the consumption of oxygen via protein interactions remaining relatively constant regardless of dose rate, the primary reaction pathway leading to a dose rate-driven change is the reaction hydroperoxyl radical (HO_2_·) with a superoxide (O_2_·^−^), which forms oxygen (O_2_) and a hydrogen peroxide anion (HO_2_–). This pathway was similarly crucial to oxygen consumption differences in pure water simulations, primarily due to lack of interactions of albumin with superoxide or hydroperoxyl radicals. Modulation of these reactions via enzymes such as SOD or a hydroperoxyl scavenger could minimize dose rate induced differences (Favaudon *et al* 2022). This increased second-order radical recombination could result in reduced DNA damage via the scavenging of the more reactive radicals.

Examining the predictions shown in figure 6 to get more insight into oxygen consumption and generation chemistry, there is a noticeable difference between CDR and UHDR radiation for reactions involving peroxyl radicals. The generation of a peroxyl radical and an interaction of two peroxyl radicals are significant factors in the dynamics of oxygen consumption. The key ranges of oxygen where a FLASH effect has been observed are *in vitro* Adrian *et al* (2020) of 1.6%–4.4% O_2_ (12–33 mmHg), and *in vivo* by Hunter *et al* between 0.7%–2.6% O_2_ (5–20 mmHg) (Tan *et al* 2023). The data provided by Adrian *et al* shows a maximal FLASH effect at their lowest oxygen level of 12 mmHg, akin to the simulated decrease in peroxyl radical formation at UHDR as seen in figure 5. This is even more prominent when compared to data from Hunter *et al* showing a more limited range in sparing induced by FLASH, which is consistent with the decrease in peroxyl formation at UHDR at lower oxygen as seen in figure 6(C).

Model simulations notably do not predict a reduction of hydrogen peroxide yield at UHDR first experimentally demonstrated by Montay-Gruel *et al* (2019) and examined under various beamline conditions there are several sources of experimental data documenting this reduction experimentally (Sunnerberg *et al* 2023b, Zhang *et al* 2024). Recent results from Rothwell *et al* show that with specific scavenging environments, specifically nitrate an aqueous electron scavenger, that this reduction is minimized and can be matched to simulations (Rothwell *et al* 2026). The model presented in this study shows a minor increase of 5% in hydrogen peroxide yield at UHDR due to additional recombination of Hydroxyl radicals as seen in figure 4. This dose rate dependance of hydrogen peroxide in simulations has been noted previously with some studies noticing up to 30% increase in yield at UHDR; this was a primary theory as to how recombination of radicals results in less biological damage (Abolfath *et al* 2020, JN *et al* 2023, Chappuis *et al* 2024, Kusumoto *et al* 2024). Matching this experimental data to simulations is still actively under investigation (Abolfath *et al* 2025, JN *et al* 2025, Tonneau *et al* 2025).

There are several simplifications used in this study that limit its interpretation to further situations. The predictions of radical changes are in solution of a singular pH with only one general protein type and no active radical scavengers such as glutathione or catalyze, as noted by Koch *et al* these can result in an order of magnitude shift in oxygen consumption (2023). These limitations are a concern when comparing to changes in biological media such as *in vitro* cellular or *in vivo* models where the FLASH effect is observed, especially between normal and tumor tissue where the microenvironment vary substantially in both scavenging capacity and pH that drastically modify the radiation chemistry (Koch *et al* 2023). Further studies analyzing these microenvironmental differences between tissues is crucial to understanding when the FLASH effect is observable to help understand its mechanism. There are various secondary parameters that could further influence these kinetics beyond varying pH, protein and addition of enzymes, these include diffusion, spatial distribution, viscosity, and overall heterogeneity of complex tissues. Still, the purpose of this work was not to fully model the FLASH effect but rather to simulate experimental data and to match the trends with oxygen and dose rate.

## 5. Conclusion

This work used a physiochemical model to simulate the time evolution of water radiolysis species under conventional and UHDR irradiation conditions, with particular emphasis on hydroxyl radical dynamics, aqueous electrons, and oxygen consumption. By matching experimental conditions, the simulations provided a mechanistic framework for interpreting observations from oxygen-depletion studies and aqueous electron measurements. Overall, the findings indicate that only a subset of proposed FLASH radiochemistry mechanisms are supported by the modeled chemical kinetics. The model predicted that radical–radical recombination contributes less than 5% to the scavenging of HO· and e_aq_^−^ even at UHDR. This limited dose-rate dependence suggests that radical quenching alone is unlikely to account for the magnitude of normal-tissue sparing observed in FLASH irradiation.

Analysis of oxygen dynamics reproduced the magnitude and dose-rate dependence of oxygen consumption reported in published *in vitro* and *in vivo* studies, supporting the robustness of the model for probing oxygen-mediated chemistry. The reaction between hydroperoxyl radical (HO_2_·) and superoxide (O_2_·^−^) emerged as the primary dose-rate sensitive pathway influencing observed oxygen consumption differences. Reduced formation of peroxyl radicals at UHDR was most pronounced within oxygen tensions (∼5–20 mmHg) corresponding to the experimentally observed oxygen ‘window’ in which FLASH sparing is most consistently reported. These results highlight peroxyl-driven chemistry as a plausible contributor to altered oxidative damage at UHDR.

Finally, the model incorporates several simplifying assumptions that constrain its applicability to cellular or *in vivo* contexts. Nonetheless, the goal of the present work was not to recreate the full biochemical complexity of the FLASH effect but to test whether radiolysis induced chemical trends with dose rate and oxygenation are consistent with experimental observations. Within this scope, the model successfully identifies specific reaction pathways that exhibit dose-rate sensitivity and provides mechanistic insight into radiochemical regimes most likely to contribute to FLASH-associated normal-tissue sparing.

## Data Availability

All data that support the findings of this study are included within the article (and any supplementary information files). Supplementary tables 1&2 available at https://doi.org/10.1088/1361-6560/aeabe5/data1.
